# Evolution of six novel ORFs in the plastome of *Mankyua chejuense* and phylogeny of eusporangiate ferns

**DOI:** 10.1038/s41598-018-34825-6

**Published:** 2018-11-07

**Authors:** Hyoung Tae Kim, Ki-Joong Kim

**Affiliations:** 1 Division of Life Sciences, School of Life Sciences, Korea University, Seoul, 02841 Korea; 2 Present Address: Institute of Agricultural Science and Technology, Chungbuk National University, Chengju, 41566 Korea

## Abstract

In this paper, three plastomes of *Mankyua chejuense, Helminthostachys zeylanica*, and *Botrychium ternatum* in Ophioglossaceae were completely sequenced in order to investigate the plastome evolution and phylogeny of eusporangiate ferns. They were similar to each other in terms of length and the gene orders; however, six unknown open reading frames (ORFs) were found between *rps4* and *trnL*-UAA genes in *M. chejuense*. Similar sequence regions of six ORFs of *M. chejuense* were found at the plastomes of *Ophioglossum californicum* and *H. zeylanica*, as well as the mitochondrial genome (mitogenome) of *H. zeylanica*, but not in *B. ternatum*. Interestingly, the translated amino acid sequences of three ORFs were more similar to the proteins of distantly related taxa such as algae and bacteria than they were to proteins in land plants. It is likely that the six ORFs region arose from endosymbiotic gene transfer (EGT) or horizontal gene transfer (HGT), but further study is needed to verify this. Phylogenetic analyses suggested that *Mankyua* was resolved as the earliest diverging lineage and that *Ophioglossum* was subsequently diverged in Ophioglossaceae. This result supports why the plastome of *M. chejuense* have contained the most ancestral six ORFs in the family.

## Introduction

Chloroplast is an apparatus for photosynthesis in plant cells that holds an independent genome compared to nuclear and mitochondrial genomes. The plastomes of land plants are typically 120–160 kb in length and have quadripartite structures^1^. Because of the strong selective constraint for photosynthesis, land plant plastomes usually contain a set of unique 100–120 photosynthetic and housekeeping genes that originate from cyanobacteria^1^. Chloroplast gene(s) or duplicated part(s) of plastomes are frequently transferred to the nuclear or mitochondrial genome (mitogenome) through intracellular gene transfer (IGT)s^2,3^. However, gene transfer to the counter direction is very rare evolutionary events^2^. Only a few cases of IGT to plastomes have been reported in unrelated plant families, such as Apiaceae^4,5^, Poaceae^6,7^, Apocynaceae^8^, and Anacardiaceae^9^. In all of these IGT cases, short portions of mitogenome were the donors to plastomes, and there has been no documented case of nuclear genome donor to plastome.

Horizontal plastome capture through hybridization between similar species is one kind of horizontal gene transfer (HGT) that is relatively common in land plants. In addition, HGT has also been documented between far distant organisms such as plant-fungus, plant-bacteria, and plant-virus^10^. Many land plants live in the symbiotic associations with fungi or bacteria. Therefore, they have relatively high chances of HGT between distant organisms. However, on the plant side, the reported HGTs were engaged in mitogenomes or nuclear genomes, not in plastomes. Land plant plastomes do not normally recombine with other genomes, therefore it is very rare for them to act as a recipient of the HGT in land plant plastomes^10^. In contrast, in the green alga *Euglena myxocylindracea*, plastomes show intron gains from bacteria^11,12^.

So far, more than 2,000 complete plastome sequences are available from public databases, such as NCBI. Plastomes, however, appear to be recalcitrant to the incorporation of foreign DNA by either IGT or HGT^9^. Only a few families, as mentioned in the previous paragraphs, have been recognized as containing DNA of nonplastome origin. However, most published reports on plastomes have been on those from seed plants. We still have limited complete plastomes for several major fern lineages. Ferns are usually divided into two groups: eusporangiate and leptosporangiate ferns. The eusporangiate ferns form basal paraphyletic assemblages because they include the eusporangiate fern clade. So far, 65 plastomes have been reported in leptosporangiate ferns^13–15^; in contrast, only nine plastomes in eusporangiate ferns have been sequenced from two species of Marattiales^16,17^, two species of Psilotales^18,19^, two species of Equisetales^18,20,21^, and one species of Ophioglossales^18^.

The order Ophioglossales of eusporangiate ferns contains a single family Ophioglossaceae, and this family consists of four genera (*Ophioglossum, Botrychium, Helmintostachys*, and *Mankyua*)^22^*. Ophioglossum* and *Botrychium* each consist of a number of species and are both relatively common in the northern hemisphere; however, both *Helmintostachys* and *Mankyua* are monotypic genera and show restricted distribution patterns in temperate regions of East Asia^23,24^. Among four genera, *Mankyua* has recently been described from a volcanic island in the Southern part of Korea as *Mankyua chejuense*^24^. It is a rare, endemic, and endangered plant species, and only a couple hundred individuals were reported to live in the specific habitats of small scattered volcanic craters called “Gotjawal” in Jeju island of Korea^25^.

In the phylogeny of Ophiglossacae, Hauk, *et al*.^26^ showed that *Ophioglossum* was the sister group to the clade of *Helminthostachys* + *Botrychium* s.l. using *rbcL* and *trnL-F* sequences. However, the phylogenetic studies including *Mankyua* have shown different phylogenetic relationships among four genera. Sun, *et al*.^27^ suggested that *M. chejuense* was sister to the clade of *Botrychium* + *Helminthostachys* and that *Ophioglossum* was the sister group to the three genera. In contrast, Shinohara, *et al*.^28^ suggested that *Botrychium* was sister to *Ophioiglossum* + *Helminthostachys* and that *Mankyua* was sister to the remaining taxa. In addition to the topological incongruences of the four genera, several nodes in the previous phylogenies of Ophioglossaceae were not strongly supported. As a result, the relationships among four genera in Ophioglossaceae remain unclear.

In this paper, plastomes of *M. chejuense, H. zeylanica*, and *B. ternatum* were completely sequenced and compared with previously reported plastomes of *O. californiacum* in order to investigate the evolution of plastomes in Ophioglossaceae. During this study, we identified approximately 10 kb insertion with six unknown ORFs between *rps4* and *trnL*-UAA genes of *M. chejuense* plastome. These six ORFs were located in the same direction as those in polycistronic genes. Therefore, we discussed the possible origins of the six ORFs through intensive comparative data analysis. In addition, the phylogeny of the four genera in the family Ophioglossaceae was reconstructed based on coding sequences of the plastome in order to resolve the enigmatic relationships among the four genera in Ophioglossaceae.

## Results and Discussion

### Genome structure and gene contents of plastomes in Ophioglossaceae

The four completely annotated plastome sequences reported in this study are available from the National Center for Biotechnology Information (NCBI) under the accession numbers of *B. ternatum* (KM817789), *H. zeylanica* (KM817788), and *M. chejuensis* 1,2 (NC017006, KP205433). The row Illumina MiSeq sequence data files also available from the NCBI database (Supplemental Table 1). We sequenced two different accessions of *M. chejuensis* using different methods: PCR-amplified Sanger sequencing and the MiSeq (Illumina, San Diego) NGS.

**Figure 1:**
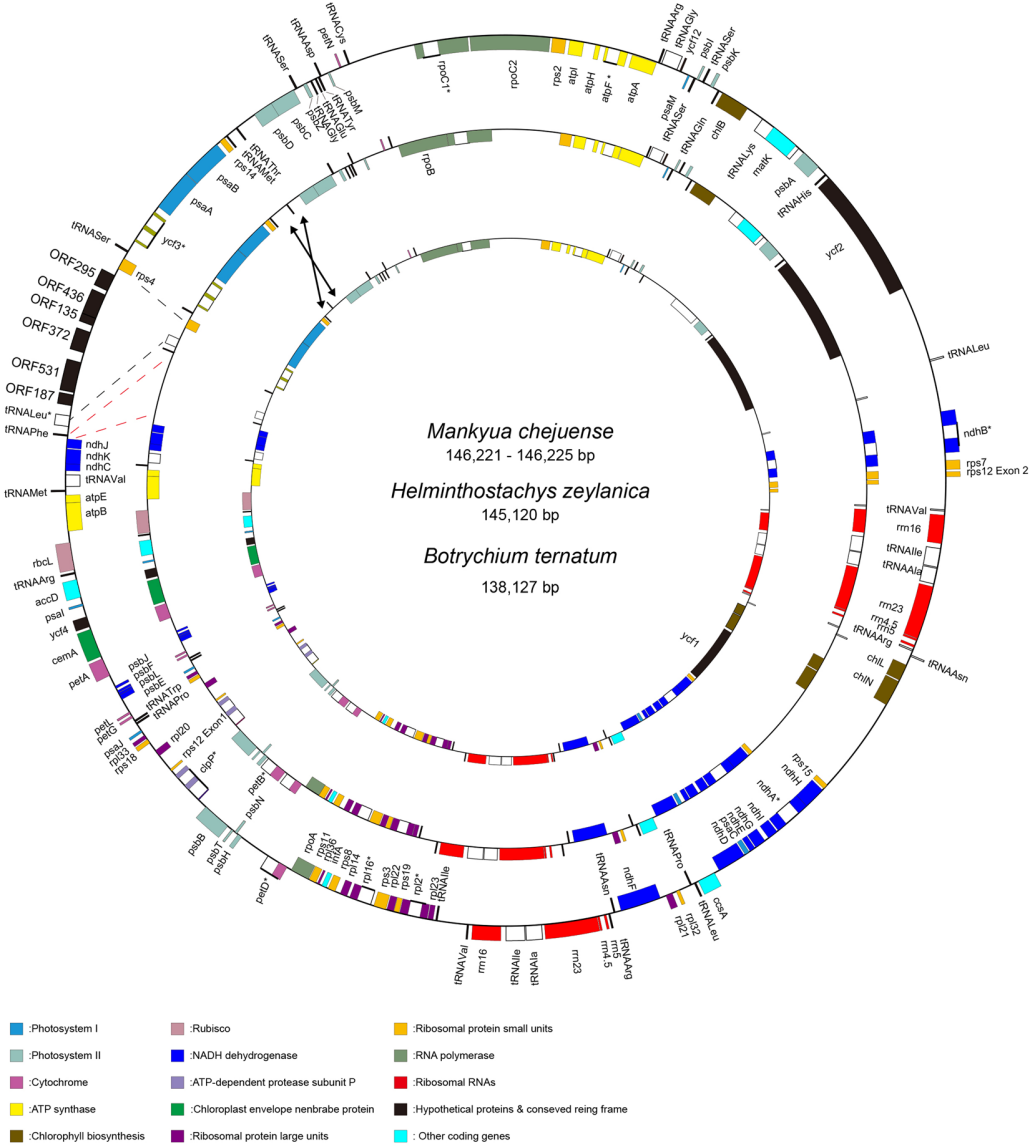
Maps of three plastomes in Ophioglossaceae. Arrows and dashes refer to inversion and expanded positions, respectively.

The plastome of *M. chejuense* sequenced through PCR was 146,221 bp in length with a large single copy (LSC) region of 106,096 bp, a small single copy (SSC) region of 20,613 bp, and two inverted repeat (IR) regions of 9,756 bp each (Fig. 1). It contained 135 genes, including 84 protein coding genes, 8 ribosomal RNAs, 37 transfer RNAs, and six unknown ORFs. Four *rRNA* and five *tRNA* genes were duplicated in the IR region (Table 1). Sixteen genes had one intron while the *clpP* and *ycf3* genes each had two introns. The plastome of *M. chejuense* sequenced through NGS was 146,225 bp. An average coverage depth of the plastome was approximately 400 times (Supplemental Table 1). A total of six poly-T length variations and 45 single nucleotide polymorphisms (SNPs) were found between two plastome sequences of *M. chejuense* (Supplementary Table S2). Thirty-seven SNPs were found at the coding regions; in particular, SNPs in *petB* and *psbB* accounted for 71% of the total SNPs. Interestingly, non-synonymous substitutions were almost three-fold the prevalence of synonymous substitutions in *petB* and *psbB*, and *petB* in plastome of *M. chejuense* sequenced by PCR method had one premature stop codon caused by substitution (TGG > TGA).

**Table 1:** Gene list of chloroplast genomes found in four genera in Ophioglossaceae.

Group of gene		Conserved genes	*M. chejuense* (PCR)	*M. chejuense* (NGS)	*H. zeylanica*	*B. ternatum*	*O. californicum*
RNA genes	Ribosomal RNAs	*rrn4.5*(x2), *rrn5*(x2), *rrn16*(x2), *rrn23*(x2)					
	Transfer RNAs	*trnA*-UGC^a^(x2), *trnC*-GCA, *trnD*-GUC, *trnE*-UUC, *trnF*-GAA, *trnfM*-CAU, *trnG*-GCC, *trnG*-UCC,* trnH*-GUG, *trnI*-CAU,* trnI*-GAU^a^(x2), *trnK*-UUU^a^,* trnL*-CAA, *trnL*-UAA^a^, *trnL*-UAG, *trnM*-CAU,* trnN*-GUU(x2), *trnP*-GGG, *trnP*-UGG, *trnQ*-UUG, *trnR*-ACG(x2),* trnR*-CCG, *trnR*-UCU,* trnS*-CGA, *trnS*-GCU, *trnS*-GGA, *trnS*-UGA,* trnT*-GGU, *trnV*-GAC(x2), *trnV*-UAC^a^, *trnW*-CCA, *trnY*-GUA			*φtrnT*-UGU	*φtrnT*-UGU	
Protein genes	Photosystem I	*psaA, psaB, psaC, psaI, psaJ, psaM*					
	Photosystem II	*psbA, psbB, psbC, psbD, psbE, psbF, psbH, psbI, psbJ, psbK, psbL, psbM, psbN, psbT, psbZ*					
	Cytochrome	*petA, petD*^a^,	*φpetB* ^a^	*petB* ^a^	*petB* ^a^	*petB* ^a^	*petB* ^a^
	ATP synthase	*atpA, atpB, atpE, atpF*^a^, *atpH, atpI*					
	Chlorophyll biosynthesis	*chlL, chlN*	*chlB*	*chlB*	*chlB*	*φchlB*	*chlB*
	Rubisco	*rbcL*					
	NADH dehydrogenease	*ndhA*^a^, *ndhB*^a^, *ndhC, ndhD, ndhE, ndhF, ndhG, ndhH, ndhI, ndhJ, ndhK*					
	ATP-dependent protease subunit P	*clpP* ^a^					
	Chloroplast envelope membrane protein	*cemA*					
Ribosomal proteins	large units	*rpl2*^a^, *rpl14, rpl16*^a^, *rpl20, rpl21, rpl22, rpl23, rpl32, rpl33, rpl36*					
	small units	*rps2, rps3, rps7, rps8, rps11, rps12*^a^, *rps14, rps15, rps18, rps19*	*rps4*	*rps4*	*rps4*	*φrps4*	*rps4*
Transcription	RNA polymerase	*rpoC1* ^a^	*rpoA, φrpoB, rpoC2*	*rpoA, φrpoB, rpoC2*	*rpoA, rpoB, φrpoC2*	*φrpoA, rpoB, φrpoC2*	*rpoA, rpoB, rpoC2*
Translation	Initiation factor	*infA*					
	Miscellaneous proteins	*accD, ccsA*	*matK*		*matK*	*φmatK*	*matK*
	Hypothetical proteins & Conserved reading frame	*ycf2, ycf3*^a^, *ycf4, ycf12*	ORF135, ORF187, ORF295, ORF372, ORF436, ORF531, *φycf1*	ORF135, ORF187, ORF295, ORF372, ORF436, ORF531, *φycf1*	*φycf1*	*ycf1*	*ycf1*

(x2): duplicated genes, ^a^: genes having introns *φ*: pseudogene.

Even though intraspecific variations of plastomes in ferns have been reported^21^, population studies of *M. chejuense* have shown extremely low genetic diversity^29^. Based on our observation of the reproduction of *M. chejuense* over three years, asexual reproduction by rhizomes was found to be very common. Therefore, these polymorphisms between two plastomes seem to not be genuine. It has been previously shown that after free-living cyanobacteria are engulfed by eukaryotes, numerous genes are translocated from plastids to nucleus^30^. Nuclear copies of organellar DNAs have frequently been found in land plants^31^, and they have led to misleading phylogeny results^32^. Consequently, PCR-amplified sequences of the plastome of *M. chejuense* might be derived from the nuclear DNA rather than the plastome of *M. chejuense*, because the nuclear copies of plastid DNA were homologous to their counterparts in the plastome and their primer regions were shared.

The plastome of *H. zeylanica* was 145,120 bp with an LSC region of 103,088 bp, an SSC of 19,950 bp, and two IR regions of 11,041 bp (Fig. 1). The plastome of *B. ternatum* was 139,127 bp with an LSC of 99,586 bp, an SSC of 20,569 bp, and two copies of IR with 9,486 bp (Fig. 1). Among four genera in Ophioglossaceae, *rps16* and *trnT-*UGU genes were commonly lost; however, pseudo *trnT*-UGU remained in the *H. zeylanica* and *B. ternatum* plastomes between *rps4* and *trnL*-UAA. The *chlB*, *matK*, *petB*, *rpoA*, *rpoB*, *rpoC2*, *rps4*, and *ycf1* were pseudogenes in at least one plastome, but not in all plastomes. But, the status of pseudogene was not confirmed because we did not study the RNA editing. In addition, there was an inversion of *trnT*-GGU in the plastome of *B. ternatum*.

### Six ORFs of *M. chejuense* and similar sequences in Ophioglossaceae

**Figure 2:**
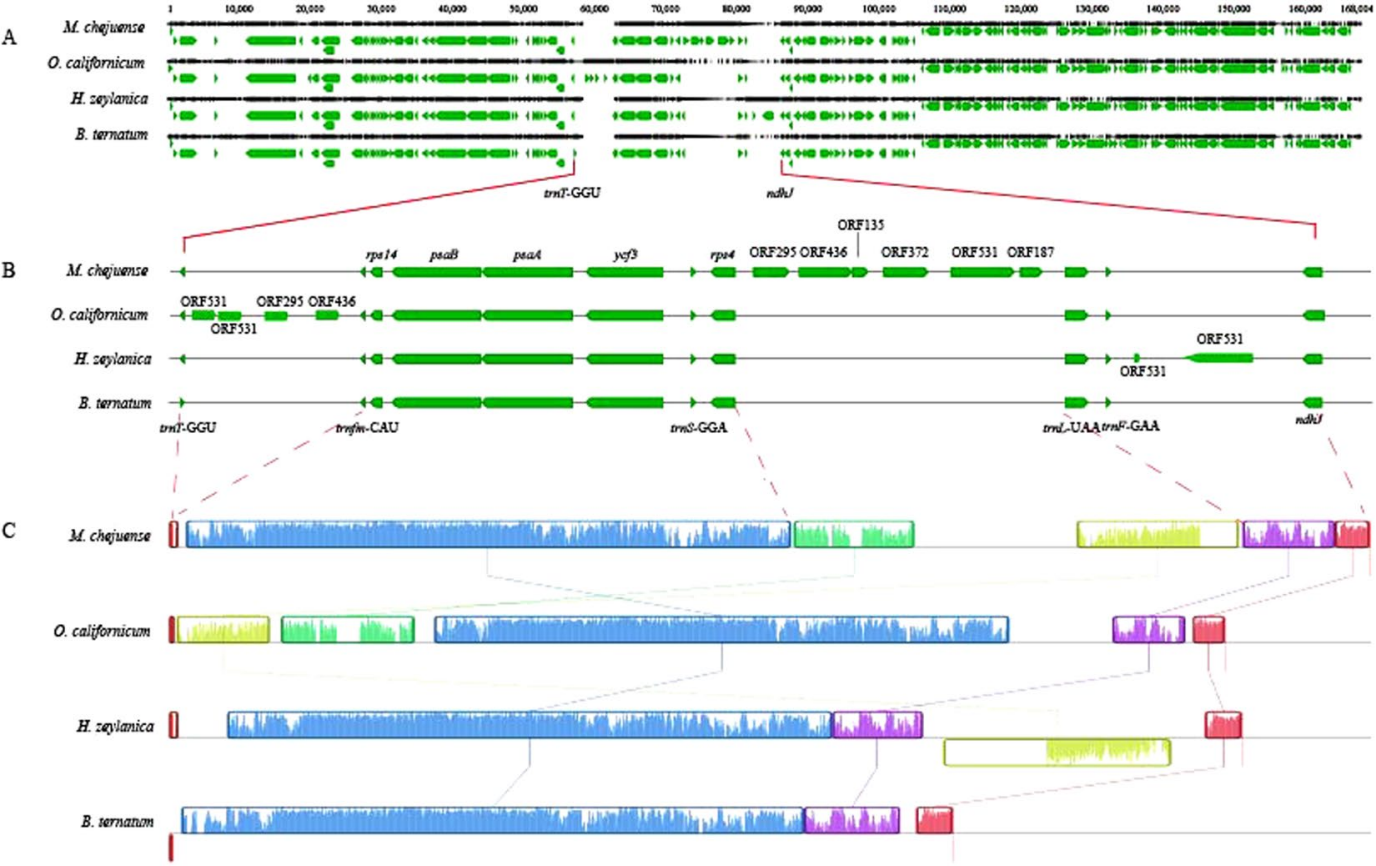
Alignments of plastomes in Ophioglossaceae. (**A**) Whole genome alignment. (**B**) Alignment between *trnT*-GGU and *ndhJ*. (**C**) Visualized alignment by MAUVE.

The most distinctive feature among the plastomes of the four genera was the region between *trnT*-GGU and *ndhJ* (Fig. 2A). Compared to *B. ternatum* plastome, the three plastomes of the other genera contained the expanded regions between *trnT*-GGU and *ndhJ*, but they were not identical (Fig. 2B). The intergenic spaces (IGSs) of *rps4* - *trnL* (*M. chejuense* and *O. californicum*), *trnT*-GGU - *trnfM*-CAU (*O. californicum* and *H. zeylanica*) and *trnF*-GAA - *ndhJ* (*H. zeylanica*) were 1.5~10 times longer than the IGSs of these regions in *B. ternatum*. In particular, six unknown ORFs (ORF295, ORF436, ORF135, ORF372, ORF531, and ORF187) were found between *rps4* and *trnL*-UAA genes in *M. chejuense*, and these six ORFs were located in the same direction as polycistronic genes. The expanded regions in *O. californicum* and *H. zeylanica* were partial of six ORFs of *M. chejuense* with structural mutations (Fig. 2C).

**Figure 3:**
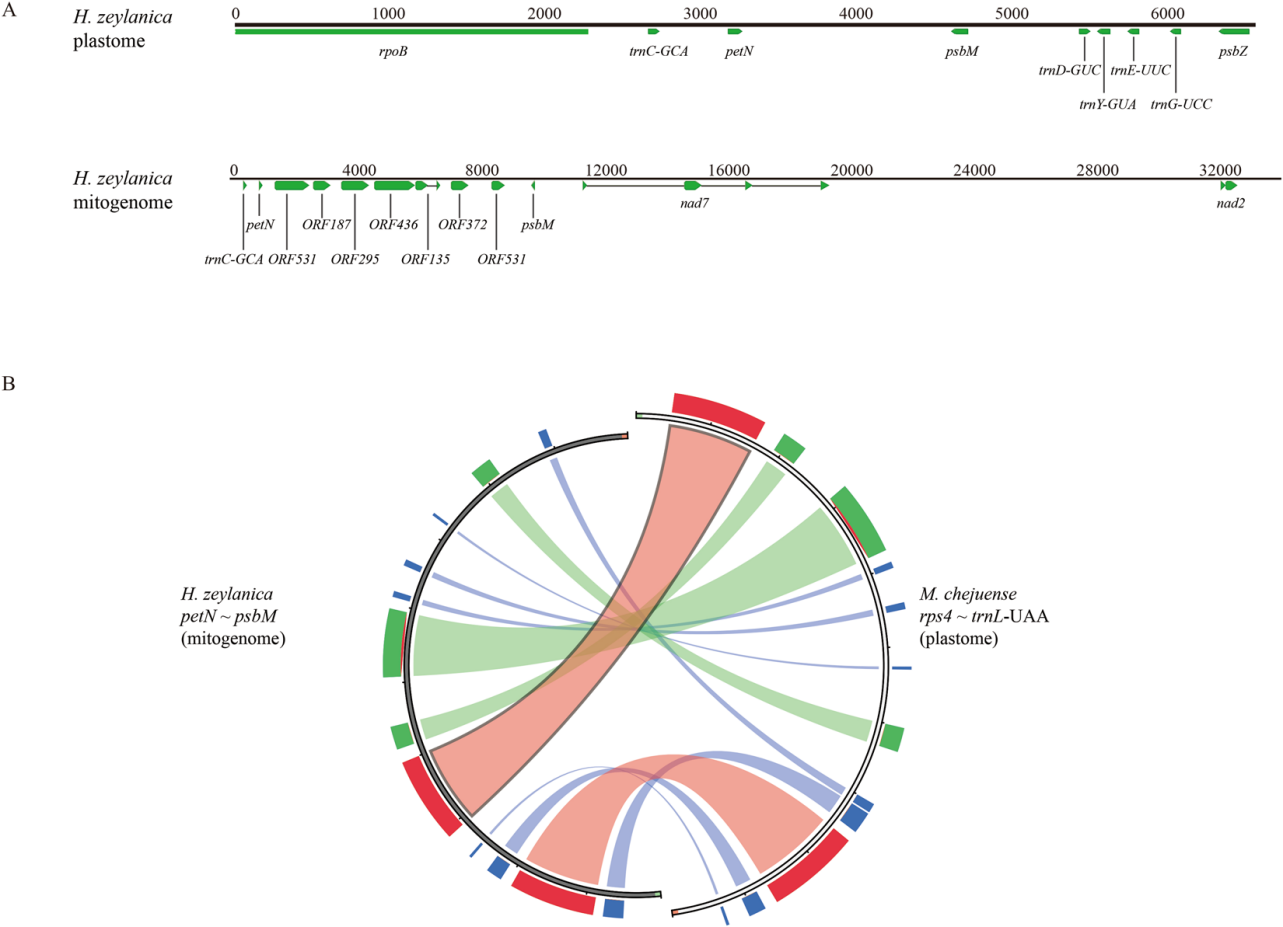
(**A**) Comparison between plastome and mitogenome of *H. zeylanica*. (**B**) Sequence similarity between six ORFs regions of *H. zeylanica* and *M. chejuense*. Score/max ratio colouring with blue < = 0.25, green < = 0.5, orange < = 0.75, and red >0.75.

Contigs generated by the de novo assembly of three sets of NGS data were hit to six ORFs region in the plastome of *M. chejuense* using blastn in order to investigate translocated ORFs into other genomes such as mitogenome. Two contigs of *H. zeylanica* with 10-11 coverage depths included sequences similar to six ORFs except for plastome contigs. One contig contained plastome genes of *petN* and *psbM* and mitogenome genes of *nad7* and *nad2* with six ORFs (Fig. 3A). IGS of *rps4* - *trnL*-UAA in the plastome of *M. chejuense* corresponded highly with *petN*-*psbM* in the mitochondrial contig of *H. zeylanica*, even though there were rearrangements and insertions/deletions (Fig. 3B). Another contig contained a region similar to ORF295 with low similarity. Certain contigs of *M. chejuense*, *H. zeylanica*, and *B. ternatum* had similar sequences of six ORFs; however, they were less than 1,500 bp with less than three coverage depths (almost around 1). These contigs were removed upon further analyses because we could not verify the assembly errors.

As the total length of NGS data of *H. zeylanica* was 4.25 Gb, and the lowest 1 C reported in Ophioglossaceae so far was 2.5 Gb^33,34^, 10-11 coverage depths implied that this contig belonged to mitogenome rather than nuclear genome, even though many plastome and mitogenome sequences were found in nuclear genomes^35^. The translated amino acid sequences of ORF135, ORF295, and ORF436 between the plastome of *M. chejuense* and mitogenome of *H. zeylanica* had over 70% identity while that of ORF372 had 40% identity (Supplementary Fig. 1). The ORF187 and ORF531 of *H. zeylanica* underwent frame-shift mutations and rearrangement, respectively.

### The origin of six ORFs

Based on blastp results (Table 2), the translated amino acid sequence of ORF295 was only similar to the protein of the green alga *Roya anglica*, which belongs to Streptophyta, and the translated amino acid sequence of ORF436 was similar to the proteins of Chlorophyta, which is a sister group of Streptophyta in Viridiplantae. Interestingly, the translated amino acid sequence of ORF531 was more similar to bacterial proteins than the proteins in Viridiplantae, even though the TrlaMp60 of *Treubia lacunosa* belonging to Streptophyta was hit to ORF531. Blastn results showed that the plastomes of very few species in ferns contained similar sequences to six ORFs (Supplementary Table S2).

**Table 2:** Results of blastp of six ORFs with e-value 10^−2^.

Gene	Description	Phylum	Species	Max.score	Total.score	Query.cover	E.value	Ident	Accession
ORF295	hypothetical protein (chloroplast)	Streptophyta	Roya anglica	89	89	65%	2.00E-17	30%	YP_009033761.1
ORF436	hypothetical protein (chloroplast)	Chlorophyta	Ettlia pseudoalveolaris	95.5	95.5	41%	3.00E-17	32%	YP_009105467.1
	hypothetical protein (chloroplast)	Chlorophyta	Prasiola crispa	68.6	68.6	42%	4.00E-09	27%	AKZ21082.1
	hypothetical protein (chloroplast)	Chlorophyta	Sarcinofilum mucosum	65.9	65.9	28%	5.00E-09	35%	YP_009367460.1
	hypothetical protein (chloroplast)	Chlorophyta	Ostreobium sp. HV05007a	67.4	67.4	28%	1.00E-08	35%	ARQ82113.1
	hypothetical protein (chloroplast)	Chlorophyta	Gloeotilopsis planctonica	67.8	67.8	37%	2.00E-08	29%	AOC61661.1
ORF531	hypothetical protein	Cyanobacteria	Pleurocapsa sp. PCC 7319	78.6	78.6	52%	2.00E-11	27%	WP_019503236.1
	hypothetical protein	Cyanobacteria	Chondrocystis sp. NIES-4102	75.1	75.1	50%	2.00E-10	27%	WP_096724718.1
	DUF3854 domain-containing protein	Cyanobacteria	Cyanothece sp. CCY0110	70.9	70.9	49%	4.00E-09	28%	WP_008277431.1
	DUF3854 domain-containing protein	Cyanobacteria	Cyanothece sp. CCY0110	70.1	70.1	49%	7.00E-09	29%	WP_008277548.1
	DUF3854 domain-containing protein	Cyanobacteria	Cyanothece sp. PCC 7822	69.3	69.3	21%	9.00E-09	36%	WP_049802779.1
	hypothetical protein	Cyanobacteria	Myxosarcina sp. GI1	69.3	69.3	50%	1.00E-08	25%	WP_052055931.1
	DUF3854 domain-containing protein	Cyanobacteria	Crocosphaera watsonii	69.3	69.3	49%	1.00E-08	29%	WP_007304689.1
	DUF3854 domain-containing protein	Cyanobacteria	Tolypothrix bouteillei	68.9	68.9	49%	2.00E-08	28%	WP_050044965.1
	hypothetical protein	Cyanobacteria	Pleurocapsa sp. CCALA 161	68.2	68.2	50%	3.00E-08	27%	WP_106238468.1
	DNA primase	Cyanobacteria	Crocosphaera watsonii WH 0402	65.5	65.5	25%	5.00E-08	36%	CCQ65996.1
	ATPase	Cyanobacteria	Aphanothece hegewaldii	67	67	48%	7.00E-08	28%	WP_106459345.1
	MULTISPECIES: DUF3854 domain-containing protein	Cyanobacteria	Cyanothece	66.2	66.2	36%	1.00E-07	31%	WP_009547941.1
	DUF3854 domain-containing protein	Cyanobacteria	Crocosphaera watsonii	66.2	66.2	50%	1.00E-07	26%	WP_007310072.1
	hypothetical protein	Cyanobacteria	Myxosarcina sp. GI1	65.9	65.9	50%	1.00E-07	25%	WP_052055870.1
	DNA primase	Cyanobacteria	Aphanothece hegewaldii	65.9	65.9	50%	2.00E-07	24%	WP_106459560.1
	hypothetical protein	Cyanobacteria	Myxosarcina sp. GI1	65.5	65.5	46%	2.00E-07	26%	WP_052056024.1
	DUF3854 domain-containing protein	Cyanobacteria	Cyanothece sp. CCY0110	64.7	64.7	34%	4.00E-07	31%	WP_008278684.1
	hypothetical protein TrlaMp60	Streptophyta	Treubia lacunosa	62.4	62.4	22%	7.00E-07	37%	YP_004927707.1
	hypothetical protein	Firmicutes	Tumebacillus sp. AR23208	61.6	61.6	24%	1.00E-06	27%	WP_087457668.1
	DUF3854 domain-containing protein	Cyanobacteria	Cyanothece sp. CCY0110	63.2	63.2	43%	1.00E-06	27%	WP_008276837.1
	hypothetical protein	Cyanobacteria	Myxosarcina sp. GI1	62.4	62.4	50%	2.00E-06	25%	WP_052056026.1
	hypothetical protein BWK76_02530	Proteobacteria	Desulfobulbaceae bacterium A2	61.6	61.6	28%	3.00E-06	30%	OQX20052.1
	hypothetical protein	Cyanobacteria	Myxosarcina sp. GI1	61.2	61.2	50%	4.00E-06	24%	WP_052055874.1
	hypothetical protein	Firmicutes	Lachnospiraceae bacterium	60.8	60.8	46%	4.00E-06	22%	WP_099450353.1
	hypothetical protein	Cyanobacteria	Myxosarcina sp. GI1	59.7	59.7	50%	1.00E-05	24%	WP_052055951.1
	hypothetical protein C7H79_02365	Proteobacteria	Nitrosomonas sp. APG5	58.5	58.5	34%	2.00E-05	31%	PSJ18450.1
	hypothetical protein	Actinobacteri	Streptomyces pini	58.5	58.5	30%	3.00E-05	25%	WP_093850669.1
	hypothetical protein	Cyanobacteria	Myxosarcina sp. GI1	58.2	58.2	50%	4.00E-05	24%	WP_052056112.1
	hypothetical protein	Actinobacteri	Mycobacterium szulgai	58.2	58.2	36%	4.00E-05	24%	WP_082965783.1
	ATP-binding protein	Actinobacteri	Streptomyces coelicolor	57.8	57.8	30%	4.00E-05	25%	WP_011030338.1
	hypothetical protein A5657_18130	Actinobacteri	Mycobacterium szulgai	57.8	57.8	36%	5.00E-05	24%	OBK51436.1
	hypothetical protein CBD94_01510	Proteobacteria	Gammaproteobacteria bacterium TMED234	55.5	55.5	49%	3.00E-04	26%	OUW91419.1
	phage/plasmid primase P4	Cyanobacteria	Stanieria cyanosphaera	54.7	54.7	50%	4.00E-04	25%	WP_015193635.1
	MULTISPECIES: hypothetical protein	Proteobacteria	Alteromonas	54.7	54.7	28%	5.00E-04	25%	WP_052010194.1
	phage P4 DNA primase domain-containing protein	Cyanobacteria	Anabaena sp. 90	53.9	53.9	52%	7.00E-04	29%	WP_015081293.1
	hypothetical protein	Cyanobacteria	Nostoc sp. ‘Peltigera malacea cyanobiont’ DB3992	53.9	53.9	30%	8.00E-04	29%	WP_099101112.1
	DNA primase	Firmicutes	Eubacterium aggregans	53.1	53.1	34%	0.001	29%	WP_090304657.1
	phage/plasmid primase P4 family C-terminal domain containing protein	Proteobacteria	Desulfovibrio africanus	53.5	53.5	20%	0.001	29%	WP_005988925.1
	primase	Firmicutes	Lactobacillus equicursoris	53.5	53.5	28%	0.001	23%	WP_008463426.1
	DNA primase	Cyanobacteria	Crocosphaera watsonii WH 0401	52.8	52.8	49%	0.001	22%	CCQ62642.1
	hypothetical protein	Proteobacteria	Thiotrichales bacterium HS_08	53.1	53.1	46%	0.001	24%	WP_103918394.1
	primase	Firmicutes	Lactobacillus delbrueckii	52.8	52.8	28%	0.002	23%	WP_003622798.1
	DUF3854 domain-containing protein	Cyanobacteria	Crocosphaera watsonii	52.4	52.4	49%	0.002	22%	WP_053074885.1
	MULTISPECIES: hypothetical protein	Proteobacteria	Defluviimonas	52.4	52.4	28%	0.002	29%	WP_035839891.1
	phage/plasmid primase P4	Proteobacteria	Mesorhizobium ciceri	51.6	51.6	25%	0.003	25%	WP_013531683.1
	hypothetical protein AMDU1_APLC00062G0028	Euryarchaeota	Thermoplasmatales archaeon A-plasma	51.6	51.6	18%	0.004	34%	EQB70371.1
	hypothetical protein BSZ19_16225	Proteobacteria	Bradyrhizobium japonicum	51.2	51.2	48%	0.004	21%	OSJ33189.1
	hypothetical protein	Cyanobacteria	Aphanizomenon flos-aquae	51.2	51.2	52%	0.004	28%	WP_027404306.1
	primase	Proteobacteria	Desulfovibrio vulgaris	51.6	51.6	22%	0.004	26%	WP_010939463.1
	primase	Proteobacteria	Desulfovibrio vulgaris	51.6	51.6	22%	0.004	26%	WP_011792015.1
	hypothetical protein A5769_14235	Actinobacteri	Mycobacterium intracellulare	51.2	51.2	11%	0.005	38%	OBG17368.1
	hypothetical protein	Actinobacteri	Mycobacterium intracellulare	51.2	51.2	11%	0.005	38%	WP_081284074.1
	hypothetical protein	Proteobacteria	Bradyrhizobium japonicum	50.8	50.8	48%	0.006	21%	WP_094184029.1
	MULTISPECIES: DNA primase	Firmicutes	Lachnospiraceae	50.8	50.8	33%	0.007	27%	WP_066730774.1
	hypothetical protein	Proteobacteria	Sandarakinorhabdus sp. AAP62	50.1	50.1	24%	0.008	22%	WP_017667662.1
	hypothetical protein	Firmicutes	Lachnospiraceae bacterium TWA4	50.4	50.4	34%	0.009	27%	WP_082039423.1
	phage/plasmid primase, P4 family	Firmicutes	Lachnospiraceae bacterium TWA4	50.1	50.1	34%	0.01	27%	KIR03447.1

The first question regarding these six ORFs is whether or not they were created through the shuffling process of endogenous sequences in the plastome. The mitogenomes of land plants rapidly evolve structurally^36^, and direct repeats and inverted repeats have served as good tools for rearrangement^37,38^. Additionally, double-strand break repairs with non-homologous end-joining affect the dynamic mitogenomic variation^39^. As a result, novel chimeric ORFs generated by the shuffling process have been reported in the mitogenomes of land plants^40,41^. However, in contrast with mitogenome, rearrangements of plastome have been generally restricted in land plants^1^, especially in eusporangiate ferns^42^. Therefore, we ruled out the possibility of the six ORFs having been generated by the shuffling process of plastome sequences.

**Figure 4:**
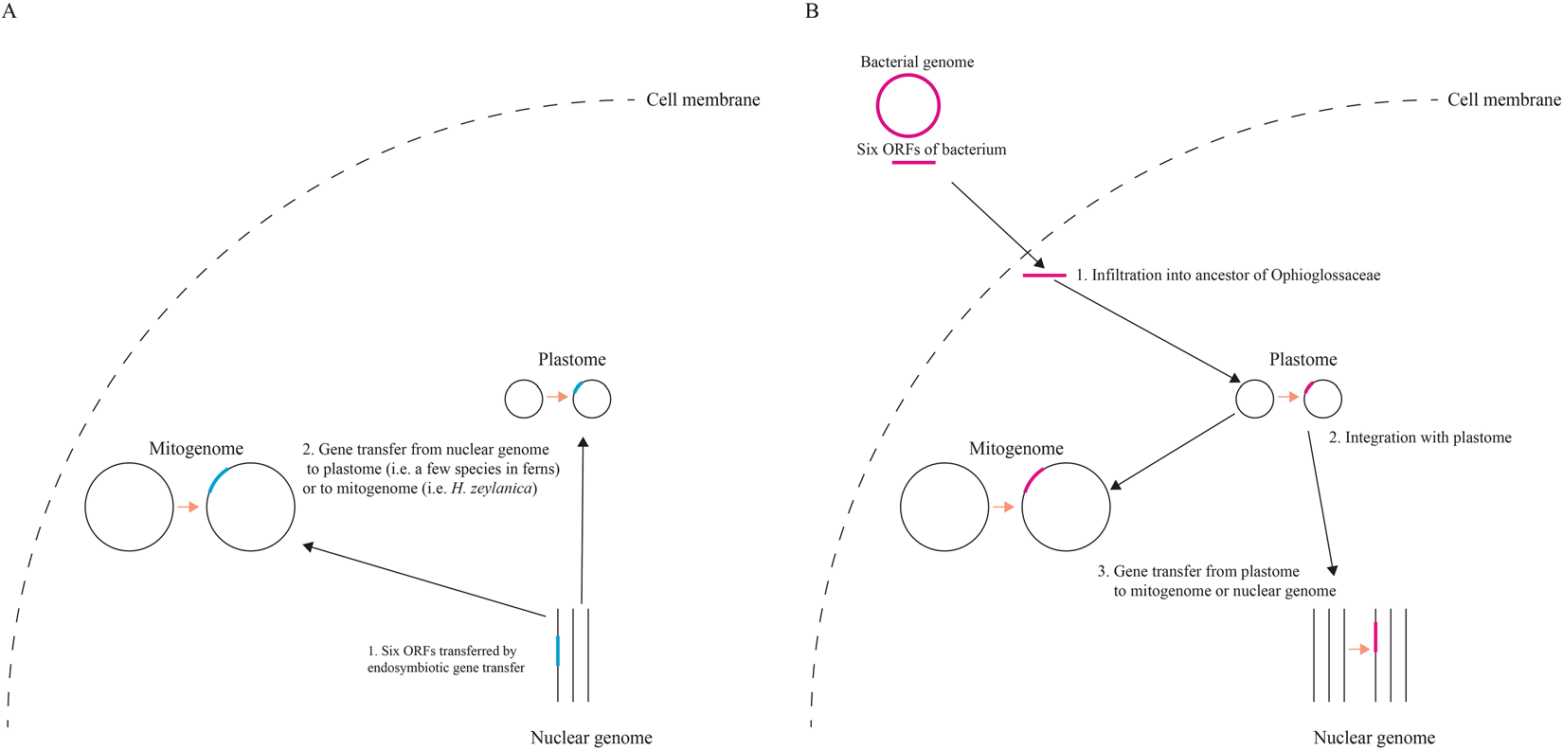
Integration models of six ORFs in Ophioglossaceae. (**A**) Ancient endosymbiotic gene transfer model. (**B**) Recent horizontal gene transfer model.

The second question is where the six ORFs region originated from prior to translocation to *M. chejuense* (or Ophioglossaceae). Gene transfer from other genomes, such as mitogenome or nuclear genome to plastome, were previously thought to occur extremely rarely if at all^43^; however, recently reported gene transfers from mitogenome to plastome^6^ have suggested the possibility of gene transfers from nuclear genome to plastome. Knox^44^ proposed that large ORFs in the plastomes of 51 species belonging to the Campanulaceae sensu lato arose from nuclear genome and Spooner, *et al*.^4^ provided the first evidence of a known nuclear sequence transferred into plastome. Martin, *et al*.^45^ revealed that massive EGTs have occurred during the evolution from plastid to nucleus in land plants. This implies that plant nuclear genome contains many genes which are orthologous to bacterial genes. Therefore, if the six ORFs region in the plastome of *M. chejuense* was transferred from the nuclear genome of *M. chejuense*, they could be similar to the ancient DNAs which remain in green algae or bacteria but not in the plastomes of land plants (Fig. 4A). In addition, as the structures of plastome in land plants have been very conserved throughout evolution with the exception of certain lineages^1^, it is likely that the translocated six ORFs region in the plastome of *M. chejuense* keeps its structure.

Another possible scenario for six ORFs is gene transfer from bacteria to the ancestor of Ophioglossaceae or directly to more ancient clade of ferns (Fig. 4B). Although a few nuclear genome sequences have been reported in land plants and most of them belonged to angiosperms^46,47^ (https://www.ncbi.nlm.nih.gov/genome), the six ORFs were only similar to the plastomes of very few fern species and the mitogenomes of *H. zeylanica*. In addition, the amino acid sequences of ORF436 and ORF531 were more similar to the genes of green algae or bacteria, which are distantly related to ferns, than to these of land plants. So far, many HGTs in land plants have been reported, and bacteria, fungi, and viruses have been agents of HGT in certain cases^48,49^. HGT from bacteria to eukaryotes has been detected in yeast^50^ along with that from bacteria to organelle^51^. Therefore, it is conceivable that the six ORFs result from HGT from bacteria to ancestor of Ophioglossaceae or more ancient clade of ferns.

### The phylogenetic relationships among genera in eusporangiate ferns

Eusporangiate ferns consist of four major families: Equisetaceae, Ophioglossaceae, Psilotaceae, and Marratiaceae. The generic relationships of eusporangiate ferns have been relatively well resolved by previous studies, except for those of the family Ophioglossaceae. Therefore, our phylogenetic study is focused on the family Ophioglossaceae. Four genera of Ophioglossaceae have different distribution patterns. Both *Botrychium* and *Ophioglossum* have cosmopolitan distributions^52^. *H. zeilanica* are distributed in Asia from India and Ceyon to South China, Taiwan, and tropical Australia^52^, but *M. chejuense* is distributed only in Jeju Island of South Korea^24^, specifically in twenty areas called “Gotjawal” created by volcanic activity. The trophophore of *Mankyua* is similar to that of *Helminthostachys*, but its sporophore is similar to that of *Ophioglossum*. In addition, *Mankyua* and *Ophioglossum* have subterranean vegetative reproduction^24^. Even though these intermediate features of *Mankyua* confused its phylogenetic position in Ophioglossaceae, phylogenetic analysis containing *Mankyua* is rare. Sun, *et al*.^27^ presented that *Ophioglossum* is the sister of *Mankyua* + *Helminthostachys* + *Botrychium* through parsimony analysis using *rbcL* data, and Shinohara, *et al*.^28^ suggested two different phylogenetic position of *Mankyua* by ML and Bayesian analysis using *rbcL* and *matK*. However, the bootstrap values for the clade comprised of more than two genera that were still under 90% according to previous studies.

**Figure 5:**
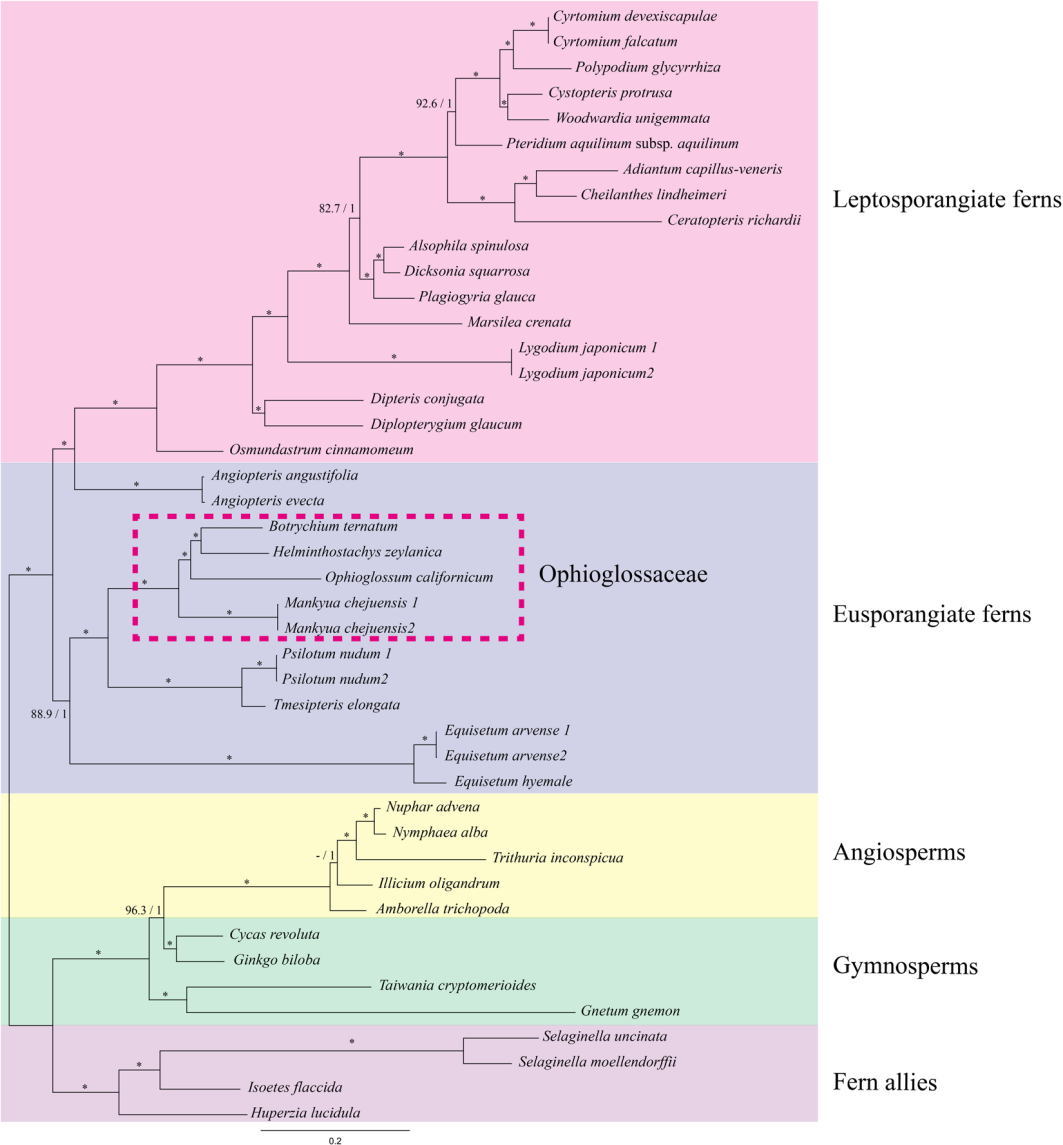
Phylogeny of eusporangiate ferns using 44 complete plastome sequences of ferns and its relatives. Numbers on the branches refer to ML bootstrap/Bayesian posterior probability. Dash and star stand for less than 50/0.5 and 100/1.0, respectively.

The phylogenetic relationships among all species used in this paper were almost identical between ML and Bayesian analysis (Fig. 5). Only the topology of ((*Amborella*, *Illicium*), (*Trithuria* (*Nuphur*, *Nymphaea*)) was supported as being stronger than that of (*Amborella* (*Illicium* (*Trithuria* (*Nuphur*, *Nymphaea*)) in terms of bootstrap value under ML analysis. Eusporangiate ferns, except for the *Angiopteris*, were monophyly with strong supports, and Ophioglossaceae also formed a clade. The phylogenetic relationships among the four genera in Ophioglossaceae in this study are completely different from those of Sun, *et al*.^27^ and Shinohara, *et al*.^28^. *Mankyua* was firstly diverged from a common ancestor of Ophioglossaceae, and then *Ophioglossum* was subsequently diverged from a common ancestor of *Helminthostachys* and *Botrychium*. Finally, *Helminthostachys* diverged from a sister group with *Botrychium*. The phylogenetic relationships of *Ophioglossum*, *Helminthostachys*, and *Botrychium* and not for *Mankyua* correspond with those described in Hauk, *et al*.^26^. They described that the ophioglossoid (*Ophioglossum* s.l.) and botrychioid (*Helminthostachys* + *Botrychium* s.l.) diverged relatively early in the evolutional history of the Ophioglossaceae.

Considering molecular phylogenetic analysis and the morphological characters of Ophioglossaceae, it seems that the ancestor of Ophioglossaceae have linear, fleshy spikes and vegetative reproduction. The ophioglossoid derived from a common ancestor have specialized their own trophophore and botrychioid have kept their trophophore and have specialized their own sporophore. In addition, the longitudinally dehiscent of sporangium in *H. zeylanica* was not plesiomorphic but apomorphic characteristic.

## Materials and Methods

### Plants materials and DNA extraction

*H. zeylanica, M. chejuense*, and *B. ternatum* were sampled at Cambodia and Jeju Island, Korea. The voucher specimens were deposited in the Korea University herbarium (KUS, K.-J. Kim *et al*., TCA2009-0806; K.-J. Jo *et al*., 2012–0028; K.-J. Kim *et al*., 2011–1638; Kim *et al*., 2012–0053). Total genomic DNA was extracted from fresh leaves using the CTAB method^53^. The DNA was purified using ultra-centrifugation in a cesium chloride/ethidium bromide gradient, then further purified by dialysis^54^.

### Sequencing of the plastome of *M. chejuense* by PCR method and assembling

The total genomic DNA of *M. chejuense* was PCR-amplified in order to construct a plastome map using a series of primer sets designed based on three plastome sequences of *Psilotum nudum, Adianthum capillus-veneris*, and *Angiopteris evecta*^16,55^. Both the long-range PCR method and normal PCR method were employed using overlapping primer sets. The PCR condition for long range amplification was as follows: initial denaturation step for 4 min at 94 °C, then 35 cycle amplifications consisting of 30 sec denaturation at 94 °C, 30 sec annealing at 53~65 °C, and about 1 min/kb extension at 68 °C, followed by an extension period of 7 min at 72 °C. The PCR condition for normal amplification was as follows: initial denaturation step for 4 min at 94 °C, then 35 cycle amplifications consisting of 30 sec denaturation at 94 °C, 30 sec annealing at 47~52 °C, and about 2 min extension at 72 °C, followed by an extension period of 3 min at 72 °C. The PCR products were purified with the MEGAquick-spin kit (iNtRON, Seoul, Korea) and the cleaned products were sequenced in both directions using an ABI 3730XL automatic sequencer. Sequence contigs were assembled using Sequencher 4.7 (Gene Code Corporation, Ann Arbor, MI, USA).

### Sequencing of the plastomes of *H. zeylanica, M. chejuense*, and *B. ternatum* by NGS and assembling

The genomic DNAs of *H. zeylanica*, *M. chejuense*, and *B. ternatum* were sequenced using MiSeq (Illumina, San Diego, CA, USA) (Supplementary Table S3). The raw reads were trimmed by trimmomatic 0.36^56^ with LEADING:10 (trimming the leading sequences until quality >10), TRAILING:10 (trimming the trailing sequences until quality >10), SLIDINGWINDOW:4:20 (trimming the window of size four for reads with the average quality less than 20), and MINLEN:50 (removing reads less than 50 bp in length). We followed the assembly method described by Kim, *et al*.^57^ using the plastome sequences of *O. californicum*^18^ and *M. chejuense* (NC017006) sequenced through PCR in this paper. Certain regions with low coverages caused by simple sequence repeats were verified using PCR.

### Gene annotation

Genes in four plastomes were annotated compared with previously reported plastomes in eusporangiate ferns based on similarity. Coding genes and tRNAs were re-checked by blastp^58^ and tRNAscan-SE^59^. ORFs were annotated using with >303 bp in length.

### Analyses of six ORFs in Ophioglossaceae

Six ORFs of *M. chejuense* were searched using blastn with 11 word size and 10^−5^ e-value and blastp with 3 word size and 10^−2^ e-value^58^ in order to investigate the homology with previously reported sequences in GenBank. In order to investigate the translocation of six ORFs into other genomes like nuclear or mitochondrial genome (mitogenome), three NGS raw data were de novo assembled using megahit^60^ and contigs were hit to six ORFs using blastn^58^. Mauve^61^ and Circoletto^62,63^ were used to visualize sequence similarity between six ORFs contigs in Ophioglossaceae.

### Phylogenetic relationships among four genera in Ophioglossaceae

The 44 complete plastome sequences of ferns and their relatives were used to resolve the unclear intergeneric relationships in Ophioglossaceae (Supplementary Table S4). Eighty-four protein coding genes were extracted from each plastome. Each gene was aligned by MAFFT^64^ and 84 aligned genes were concatenated into a single aligned sequence.

The best-fit nucleotide substitution models for each gene position in a single concatenated sequence were evaluated using Partitionfinder V2.1.1^65,66^. The maximum likelihood (ML) analysis was inferred by RAxML Black Box^67^ in CIPRES Science Gateway^68^ and Bayesian inference (BI) analysis was inferred by Mrbayes^69^ under GTR substitution model with gamma-distributed rate variation and a proportion of invariable sites (ngen = 1,000,000, samplefreq = 200, burninfrac = 0.25).

## Electronic supplementary material

Supplementary Dataset 1

## Data Availability

The complete sequence data generated during and/or analyzed during the current study are available in the NCBI GenBank repository. All data generated or analyzed during this study are included in this published article and its Supplementary Information files.
