# Supplementary material for: Evolution of six novel ORFs in the plastome of Mankyua chejuense and phylogeny of eusporangiate ferns
Source: Sci Rep. 2018 Nov 7;8:16466. doi: 10.1038/s41598-018-34825-6 (PMC6220310; doi:10.1038/s41598-018-34825-6)
Supplement: Supplementary file 1 — Supplementary Dataset 1 [file 41598_2018_34825_MOESM1_ESM.pdf]

Supplementary Information

Evolution of six novel ORFs in the plastome of *Mankyua chejuense* and phylogeny of eusporangiate ferns

Hyoung Tae Kim[1,2] and Ki-Joong Kim[1*]

[1] Division of Life Sciences, School of Life Sciences, Korea University, Seoul 02841, Korea

[2] Current address: Institute of Agricultural Science and Technology, Chungbuk National University, Chengju 41566, Korea

*Corresponding author: E-mail: kimkj@korea.ac.kr

SUPPLEMENTARY Tables

Supplementary Table 1. Summary of MiSeq data of three species in Ophioglossaceae

Taxa	Total number of reads	Number of reads after trimming	Average length of read after trimming (bp)	# of mapped reads	% of mapped reads	Coverage depth	SRA accession
Mankyua chejuensis	14,559,808	12,296,222	259.5	211,964	1.72 %	393.6	SRR7630500
Helminthostachys zeylanica	15,929,318	14,644,510	281.7	196,914	1.34 %	393.9	SRR7630499
Botrychium ternatum	15,640,592	14,133,728	276.3	205,530	1.45 %	398.0	SRR7630501

Supplementary Table 2. Base substitutions between two plastome sequences of *M. chejuense*.

Type	Region	Coding region		Noncoding region	Total
		Synonymous substitution	Non-synonymous substitution		
Noncoding region	intergenic space			4	8
	petB intron			3	
	rpoC1 intron			1	
Coding region	*petB*	4	11		37
	psbB	4	13		
	ORF436		2		
	ORF531		1		
	chlL	1			
	φycf1		1		
Total		9	28	8	45

φ: pseudogene

Supplementary Table 3. Results of blastn of six ORFs with e-value 10^{-5}.

Gene	Species	Max score	Total score	Query cover	E value	Identity	Accession
ORF295	*Ophioglossum californicum*	127	127	49%	3.00E-25	67%	KC117178.1
ORF436	*Ophioglossum californicum*	150	150	25%	2.00E-32	70%	KC117178.1
	Equisetum arvense	68	136	9%	2.00E-07	73%	N968380.1
ORF531	*Helminthostachys zeylanica*	921	921	94%	0	74%	KM817788.2
	Ophioglossum californicum	203	383	68%	4.00E-48	70%	KC117178.1
	Lepisorus clathratus	131	262	20%	2.00E-26	69%	KY419704.1
	Angiopteris evecta	102	204	14%	1.00E-17	71%	DQ821119.1

Supplementary Table 4. The list of 31 ferns, four fern allies, four gymnosperms and five angiosperms for phylogenetic analysis.

Group	Order	Species	Accession
**Fern allies (4)**	Isotales	*Isoetes flaccida*	NC_014675
	Lycopodiales	*Huperzia lucidula*	NC_006861
	Selaginellales	*Selaginella moellendorffii*	NC_013086
	Selaginellales	*Selaginella uncinata*	AB197035
**Ferns (31)**	Equisetales	*Equisetum arvense* 1	NC_014699
	Equisetales	*Equisetum arvense* 2	JN968380
	Equisetales	*Equisetum hyemale*	NC_020146
	Psilotales	*Psilotum nudum* 1	NC_003386
	Psilotales	*Psilotum nudum* 2	KC117179
	Psilotales	*Tmesipteris elongata*	KJ569699
	Ophioglossales	*Botrychium ternatum* *	KM817789
	Ophioglossales	*Helminthostachys zeylanica* *	KM817788
	Ophioglossales	*Mankyua chejuensis* 1 *	NC_017006
	Ophioglossales	*Mankyua chejuensis* 2 *	KP205433
	Ophioglossales	*Ophioglossum californicum*	NC_020147
	Marattiales	*Angiopteris angustifolia*	NC_026300
	Marattiales	*Angiopteris evecta*	NC_008829
	Osmundales	*Osmundastrum cinnamomeum*	NC_024157
	Gleicheniales	*Diplopterygium glaucum*	NC_024158
	Gleicheniales	*Dipteris conjugata*	KP136829
	Schizaeales	*Lygodium japonicum* 1	NC_022136
	Schizaeales	*Lygodium japonicum* 2	NC_022136
	Salviniales	*Marsilea crenata*	NC_022137
	Cyatheales	*Alsophila spinulosa*	NC_012818
	Cyatheales	*Dicksonia squarrosa*	KJ569698
	Cyatheales	*Plagiogyria glauca*	KP136831
	Polypodiales	*Adiantum capillus-veneris*	NC_004766
	Polypodiales	*Ceratopteris richardii*	KM052729
	Polypodiales	*Cheilanthes lindheimeri*	NC_014592
	Polypodiales	*Cyrtomium devexiscapulae*	NC_028542
	Polypodiales	*Cyrtomium falcatum*	NC_028705
	Polypodiales	*Cystopteris protrusa*	KP136830
	Polypodiales	*Polypodium glycyrrhiza*	KP136832
	Polypodiales	*Pteridium aquilinum subsp. aquilinum*	NC_014348
	Polypodiales	*Woodwardia unigemmata*	NC_028543
**Gymnosperms (4)**	Cycadales	*Cycas revoluta*	NC_020319
	Ginkgoales	*Ginkgo biloba*	NC_016986
	Cupressales	*Taiwania cryptomerioides*	NC_016065
	Gnetales	*Gnetum gnemon*	NC_026301
**Angiosperms (5)**	Amborellales	*Amborella trichopoda*	NC_005086
	Austrobaileyales	*Illicium oligandrum*	NC_009600
	Nymphaeales	*Nuphar advena*	NC_008788
	Nymphaeales	*Nymphaea alba*	NC_006050
	Nymphaeales	*Trithuria inconspicua*	NC_020372

* indicates the plastome sequences reported in this study

SUPPLEMENTARY FIGURE

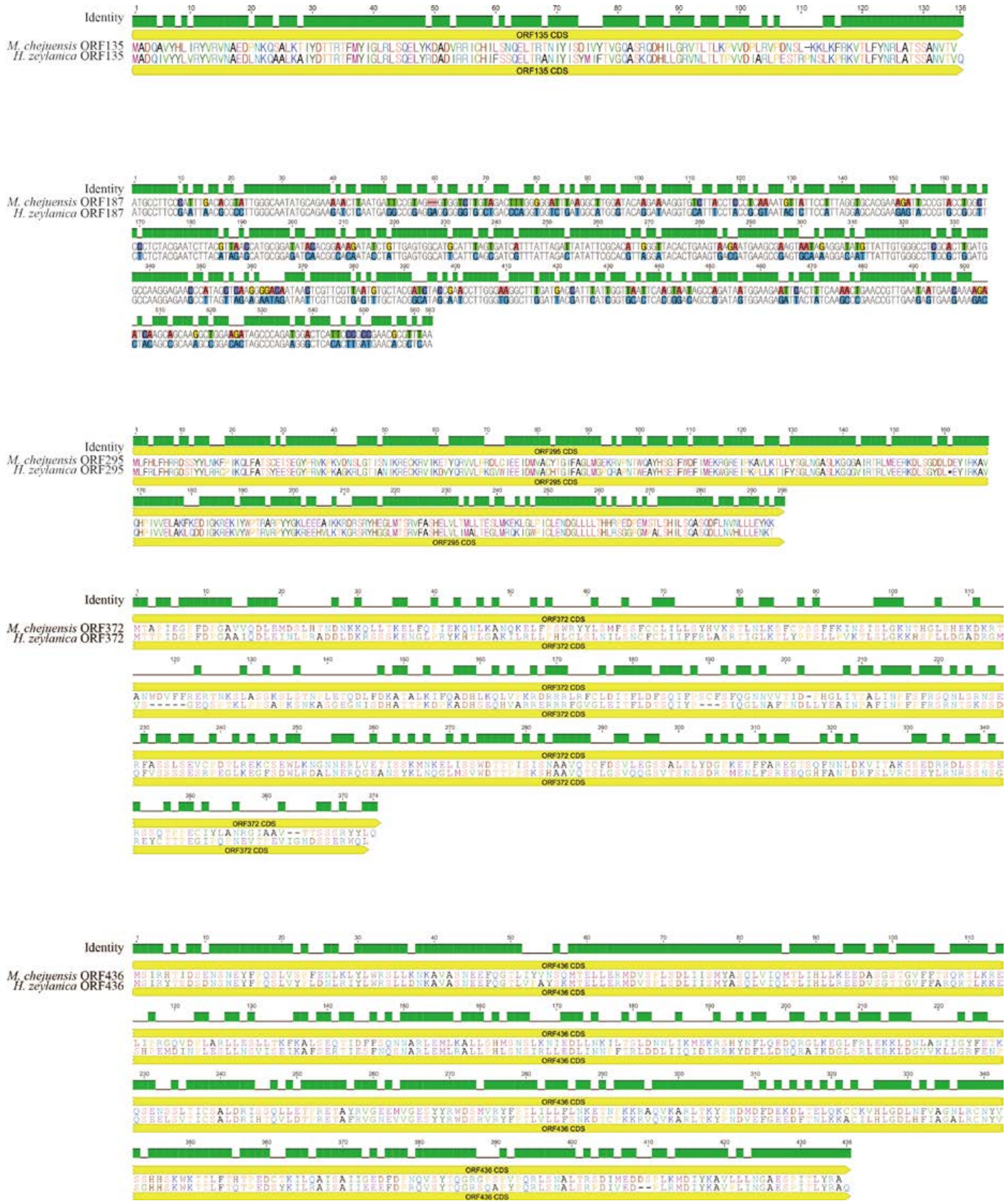

Figure S1. Alignments of six ORFs amino acid sequences between *M. chejuense* and *H. zeylanica*.
